# Malaria rapid diagnostic tests: *Plasmodium falciparum *infections with high parasite densities may generate false positive *Plasmodium vivax *pLDH lines

**DOI:** 10.1186/1475-2875-9-198

**Published:** 2010-07-10

**Authors:** Jessica Maltha, Philippe Gillet, Lieselotte Cnops, Jef van den Ende, Marjan van Esbroeck, Jan Jacobs

**Affiliations:** 1 Faculty of Health, Medicine and Life Sciences (FHML), Maastricht, The Netherlands; 2 Department of Clinical Sciences, Institute of Tropical Medicine (ITM), Unit of Tropical Laboratory Medicine, Nationalestraat 155, B 2000 Antwerp, Belgium

## Abstract

**Background:**

Most malaria rapid diagnostic tests (RDTs) detect *Plasmodium falciparum *and an antigen common to the four species. *Plasmodium vivax*-specific RDTs target *P. vivax-*specific parasite lactate dehydrogenase (Pv-pLDH). Previous observations of false positive Pv-pLDH test lines in *P. falciparum *samples incited to the present study, which assessed *P. vivax*-specific RDTs for the occurrence of false positive Pv-pLDH lines in *P. falciparum *samples.

**Methods:**

Nine *P. vivax*-specific RDTs were tested with 85 *P. falciparum *samples of high (≥2%) parasite density. Mixed *P. falciparum/P. vivax *infections were ruled out by real-time PCR. The RDTs included two-band (detecting Pv-pLDH), three-band (detecting *P. falciparum*-antigen and Pv-pLDH) and four-band RDTs (detecting *P. falciparum*, Pv-pLDH and pan-pLDH).

**Results:**

False positive Pv-pLDH lines were observed in 6/9 RDTs (including two- three- and four-band RDTs). They occurred in the individual RDT brands at frequencies ranging from 8.2% to 29.1%. For 19/85 samples, at least two RDT brands generated a false positive Pv-pLDH line. Sixteen of 85 (18.8%) false positive lines were of medium or strong line intensity. There was no significant relation between false positive results and parasite density or geographic origin of the samples.

**Conclusion:**

False positive Pv-pLDH lines in *P. falciparum *samples with high parasite density occurred in 6/9 *P. vivax-*specific RDTs. This is of concern as *P. falciparum *and *P. vivax *are co-circulating in many regions. The diagnosis of life-threatening *P. falciparum *malaria may be missed (two-band Pv-pLDH RDT), or the patient may be treated incorrectly with primaquine (three- or four-band RDTs).

## Background

Malaria rapid diagnostic tests (RDTs) are immunochromatographic tests targeting antigens of one or more *Plasmodium *species. Signals are visible as cherry-red to purple coloured lines, comprising a control line (which indicates that the test has been performed well) and one or two test lines. The initially developed two band tests generate a test line that targets *P. falciparum *by detecting either histidine-rich protein 2 (HRP-2) or *P. falciparum*-specific parasite lactate dehydrogenase (Pf-pLDH). The later developed three band tests include a second target that is common to the four *Plasmodium *species, such as aldolase or pan-specific parasite lactate dehydrogenase (pan-pLDH). However, the conventional three-band RDTs, detecting a *P. falciparum*-specific antigen and a pan-*Plasmodium *antigen, cannot distinguish between a *P. falciparum *infection and a mixed infection with *P. vivax *when both test lines are observed. Differentiation between the non-*falciparum *species is neither possible. *P. falciparum *and *P. vivax *infections require different treatment, which makes discrimination between the two species important. RDTs specific to *P. vivax *could be useful. There are two-band RDTs that detect *Plasmodium vivax*-specific pLDH (Pv-pLDH), three-band RDTs in which Pv-pLDH is combined with HRP-2 or Pf-pLDH, and so-called four-band tests that combine detection of HRP-2, pan-pLDH and Pv-pLDH. The Pv-pLDH tests have undergone limited evaluation [1-3].

In previous evaluations of RDTs targeting Pv-pLDH, rare but consistent false positive Pv-pLDH test lines were observed among *P. falciparum *samples, especially at high parasite densities [2-4]. These observations are of concern as this limits the potential use of the Pv-pLDH directed RDTs, both in endemic and non-endemic settings. In order to study the extent of this phenomenon among other RDTs, the present study was undertaken to challenge available RDT brands with a Pv-pLDH test line to a panel of *P. falciparum *samples with high parasite densities.

## Methods

### Study design

Several RDTs were retrospectively evaluated in a reference laboratory on a panel of stored whole blood samples obtained in patients suspected of malaria. The reference method was microscopy corrected by polymerase chain reaction (PCR).

### Patient samples

In this study stored whole blood samples (-70°C) were used, obtained in returned international travelers suspected of malaria presenting at the outpatient department of the Institute of Tropical Medicine (ITM), Antwerp, Belgium, or submitted by other Belgian laboratories to ITM in its function of National Reference Center. Samples had been obtained between 1996 and 2009 and were classified in regions of travel destinations according to the United Nations classification of geographical region and composition [5]. All samples were evaluated by microscopy and real-time PCR for species identification (ruling out mixed infections) and determination of parasite density, as described previously [6,7]. For the purpose of this study, the more convenient parasite density expressed by % of infected red blood cells was applied, thereby assuming 50,000/μl to be equal to 1% of red blood cells parasitized [8]. *P. falciparum*-infected samples with parasite densities ≥2% (≥100,000/μl) were selected.

### Malaria rapid diagnostic tests

RDTs containing a *P. vivax*-specific test line were selected, including those published on the World Health Organization (WHO) list of RDT manufacturers with adequate evidence of good manufacturing [9] as well as others available on the international market. We checked the package inserts to ensure the antigen used was *P. vivax*-specific. In line with other comparative evaluations [7,10] it was decided not to display individual RDT brand names because of the wide lot-to-lot variability and the frequent changes in composition and brand names and types [1].

### Test procedures

Tests were performed according to the manufacturers' instructions, except that a pipette (Finnpipette, Helsinki, Finland) was used instead of the transfer devices supplied by the manufacturer. The laboratory technicians involved in the study have received a detailed training and their performance and agreement are monitored by participation to internal and external quality control assessments. Readings were done by three subsequent observers, of whom the first always was the one performing the test, and carried out at daylight assisted by an electric bulb. The observers were blinded to each others readings and to the results of microscopy and PCR. In case no control line was observed the test was considered invalid and repeated. Test line intensities were scored according to a system of five categories as described previously [6]: none (no line visible), faint (barely visible line), weak (paler than the control line), medium (equal to the control line) and strong (stronger than the control line). Test results were based on consensus agreement: the same test result observed by at least two out of three readers. In case of no consensus the result of the first reader was considered. Pv-pLDH lines generated by *P. falciparum *samples will be further referred to as false positive Pv-pLDH lines.

### Statistical analysis

The nonparametric Spearman's rank correlation coefficient *r*_*s *_was used to measure the strength of association between parasite density and the number of RDTs with a false positive Pv-pLDH test line. Associations were considered significant at a p-value < 0.05. Inter-observer agreement for line intensities and positive and negative test results were expressed by kappa values for each pair of observers and by the percentage of overall agreement between the three observers.

### Ethical review

The study was reviewed and approved by the Institutional Review Board of ITM and by the Ethical Committee of Antwerp University, Belgium.

## Results

### Collection of samples and RDTs

Eighty-five *P. falciparum *samples with a parasite density ≥2% (100,000/μl) were selected. The parasite densities ranged from 2-35%, with 30 samples of at least 20% parasite density.

The male:female ratio was 3:1, with a median age of 40 years (range 3-98 years) and three children were under the age of five years. Samples were obtained in West Africa (n = 34), Middle Africa (n = 22), East Africa (n = 5), Southern Africa (n = 4) and West Asia (n = 1). Of 19 samples no data on the geographic origin were known and could not be retrieved. Four RDT brands (numbers. 1, 4, 7 and 9) were tested with less than 85 samples, due to either a lack of RDTs or a lack of sample.

Twenty different RDT brands were selected. Although their product name referred to *P. vivax*-specificity (for instance, by adding the epithet "Pf/Pv"), two of them in fact targeted pan-pLDH instead of Pv-pLDH: these RDTs were not considered for evaluation. Seven companies marketing eight RDT brands did not reply to the order of RDTs, despite several reminders via email contact. One *P. vivax*-specific three-band RDT was not included in this study because of bad clearance of the background, which made reading results impossible. The final panel consisted of nine different RDT brands from seven manufacturers, including one two-band (single Pv-pLDH test line), three three-band (Pv-pLDH and HRP-2 test line) and five four-band RDTs (Pv-pLDH, HRP-2 and pan-pLDH test line). Five RDTs had CE mark compliance and two were included in the WHO list of good manufacturing practices.

Three different four-band RDT brands (numbers 6, 7 and 8) had similar package inserts and the cassettes had identical morphology. Two three-band RDTs (numbers. 2 and 3) also had identical cassettes and similar package inserts. RDT numbers 1 and 4 were from the same manufacturer, as well as RDT numbers 2 and 5.

### Test characteristics

There were no invalid test results. As expected, all the *P. falciparum *samples showed positive test lines for the HRP-2 and the pan-pLDH test lines, if present on the cassette. Table 1 lists the results for the Pv-pLDH test lines, expressed by line intensities. In total there were 85 false positive Pv-pLDH lines in six RDT brands, caused by a total of 42 samples. In the individual RDT brands they occurred at frequencies of 8.2% (7/85 samples) up to 29.1% (23/79 samples), among two-, three- and four-band tests. There was no difference between RDTs that were CE-marked or WHO-listed and those which were not.

**Table 1:** Line intensity reading for Pv-pLDH lines in *P. falciparum *samples with parasite densities ≥2%

			Line intensity readings, number of samples				
				
Nr	Type	Nr of samples tested	Faint	Weak	Medium	Strong	Total positive (% of total samples)
1	Two-band	84	7	4	1	4	16 (19.0%)
2	Three-band	85					0
3	Three-band	85					0
4	Three-band	66	2		5	4	11 (16.7%)
5	Four-band	85					0
6	Four-band	85	8	13			21 (24.7%)
7	Four-band	79	19	3	1		23 (29.1%)
8	Four-band	85	2	5			7 (8.2%)
9	Four-band	82	1	5	1		7 (8.5%)

Two-band: Pv-pLDH test line; Three-band: Pv-pLDH and HRP-2 test line; Four-band: Pv-pLDH, HRP-2 and pan-pLDH test line.

Table 2 lists the details of consensus readings of line intensities for the false positive Pv-pLDH test lines, according to parasite densities and region of infection. In 2/736 readings there was no consensus and the results of the first reader were considered. Nineteen samples generated a false positive line in at least two RDTs. There was no apparent relation between parasite density of samples and the occurrence of false positive Pv-pLDH lines (*r*_s _= 0,155, p = 0,153). False-positive Pv-pLDH lines occurred exclusively in samples from patients returning from Middle or West Africa (Table 3), but there was no significant relation between geographic origin of samples and false-positive results.

**Table 2:** False positive Pv-pLDH lines in 42 *P. falciparum *samples with parasite densities ≥2%

Samples			RDT number and type						
		
Nr	% parasite density	Origin	1. Two-band	4. Three-band	6. Four-band	7. Four-band	8. Four-band	9. Four-band	Total RDTs positive
1	2,0	MAF	-	-	-	-	-	W	1
2	2,2	MAF	-	*	F	-	-	-	1
3	2,3	MAF	-	*	W	-	-	-	1
4	2,8	WAF	F	F	-	-	-	-	2
5	3,2	MAF	W	S	W	W	W	W	6
6	3,2	MAF	-	*	-	F	F	-	2
7	3,3	WAF	-	*	F	-	-	-	1
8	3,7	ND	S	*	-	F	-	-	2
9	3,8	ND	-	-	-	F	-	-	1
10	3,9	ND	-	-	W	F	-	-	2
11	4,0	WAF	S	S	W	F	W	-	5
12	4,2	WAF	W	M	-	-	-	-	2
13	4,5	WAF	F	F	-	-	-	-	2
14	4,6	MAF	-	-	F	-	-	-	1
15	5,3	WAF	M	M	W	F	-	-	4
16	5,5	WAF	-	-	-	F	-	-	1
17	5,7	MAF	S	S	W	W	-	F	5
18	6,6	MAF	F	*	-	-	-	-	1
19	7,0	ND	-	-	W	F	-	-	2
20	7,2	WAF	S	*	W	M	W	M	5
21	7,4	WAF	-	-	-	F	-	-	1
22	7,6	MAF	-	-	-	F	-	-	1
23	9,1	ND	-	-	-	-	-	W	1
24	9,6	ND	*	M	W	F	-	-	3
25	20,0	ND	-	-	-	F	-	-	1
26	20,0	WAF	F	-	-	F	-	-	2
27	20,0	WAF	-	-	W	-	-	-	1
28	20,0	WAF	W	S	W	F	W	-	5
29	20,0	WAF	W	M	F	F	-	W	5
30	20,0	WAF	-	-	F	F	-	-	2
31	20,0	ND	F	-	-	-	-	-	1
32	20,0	ND	F	-	-	F	-	-	2
33	20,0	WAF	-	-	-	F	-	-	1
34	20,0	MAF	-	*	-	-	-	W	1
35	20,0	ND	-	*	F	-	-	-	1
36	20,0	MAF	F	*	-	-	-	-	1
37	20,0	WAF	-	*	W	-	-	-	1
38	20,0	WAF	-	*	F	-	-	-	1
39	20,0	WAF	-	*	F	-	-	-	1
40	20,0	MAF	-	-	-	F	-	-	1
41	20,0	WAF	-	M	W	W	W	-	4
42	35,0	WAF	-	*	-	-	F	-	1
							
Total false positive Pv-pLDH lines			16	11	21	23	7	7	85

MAF = Middle Africa; WAF = West Africa; ND = No Data

F = faint; W = weak; M = medium; S = strong; - = negative; * = sample not tested due to lack of RDT or sample

**Table 3:** Numbers of *P. falciparum *samples generating false positive Pv-pLDH lines according to geographic origin

	Numbers of samples tested*		
	
Origin (region)	Total	Negative	Positive** (% of total samples)
East Africa	5	5	0
Middle Africa	22	10	12 (54.5)
Southern Africa	4	4	0
West Africa	34	14	20 (58.8)
West Asia	1	1	0
No data	19	9	10 (52.6)
			
All samples	85	43	42 (49.4)

* Nine RDT brands were tested with a panel of 85 samples

** Defined as a sample with a false positive Pv-pLDH line in ≥1 RDT brand

Most (69/85, 81.2%) false-positive Pv-pLDH readings were faint or weak (Table 1), but strong line intensities were observed in two RDT brands (Tables 1 and 2). Taken all readings together, inter-observer agreements for line intensity readings were good for HRP-2, pan-pLDH and Pv-pLDH test lines. For the Pv-pLDH line, in terms of positive and negative readings for all brands together, kappa values between pairs of observers were good (0.79, 0.70, 0.77) and overall agreement was excellent (88.0%). For the RDT brands considered separately, overall agreement for the Pv-pLDH line ranged from 75.9% to 100%.

## Discussion

In this study, six out of nine *P. vivax-*specific RDTs showed false positive Pv-pLDH lines when challenged to a panel of 85 *P. falciparum *samples with high (≥2%) parasite densities, in which mixed infections with *P. vivax *were excluded by PCR analysis. Frequencies for individual brands ranged from 8.2% (7/85 samples) to 29.1% (23/79 samples).

*Plasmodium vivax *accounts for almost half of the malaria infections worldwide and is no longer considered as a mild infection: complicated infections have been demonstrated in both endemic countries and in returned travelers [11,12]. In addition, *P. vivax *malaria may be chloroquine resistant and has a tendency to relapse [13]. To eradicate the dormant liver stages, primaquine treatment is needed. Primaquine is contraindicated in case of glucose-6-phosphate dehydrogenase (G6PD) deficiency, due to the risk of hemolysis [14,15]. G6PD deficiency is common in most *P. vivax *malaria areas; moreover, in these areas G6PD testing is impractical due to a lack of funds, equipments or expertise [16].

RDTs detecting *P. vivax*-specific pLDH are of additional value for the diagnosis of malaria in both *P. vivax *endemic areas and in the setting of travel medicine. The conventional three-band RDTs detecting a *P. falciparum*-specific antigen and a pan-*Plasmodium *antigen cannot distinguish between a *P. falciparum *infection and a mixed infection with *P. vivax *when both test lines are observed. Differentiation between the non-*falciparum *species is neither possible. Three- or four-band RDTs that target Pv-pLDH have the advantage that they can detect *P. vivax *in mixed infections: they are an adjunct to microscopy as *P. vivax *is often microscopically under diagnosed in mixed infections [13]. In addition they can be used to distinguish between *Plasmodium ovale *and *P. vivax *[2]. This is an advantage in the non-endemic setting, where microscopic differentiation between *P. ovale *and *P. vivax *is notoriously difficult [17].

*Plasmodium vivax*-specific RDTs have hardly been evaluated [18-22], and are not discussed in any of the recent reviews on malaria RDTs [1,8,17,23]. The present findings are of concern particularly in areas where *P. falciparum *and *P. vivax *are co-circulating: in case of a two-band Pv-pLDH test, the diagnosis of life-threatening *P. falciparum *malaria may be missed, which is especially of concern in remote areas among less experienced staff and without backup of microscopy. In case of a three- or four-band RDT, the patient may be treated incorrectly with primaquine, leading to severe hemolysis in patients with G6PD-deficiency.

Among the limitations of the present study, we should mention its retrospective nature (precluding further work-up of samples), the test conditions in a reference laboratory (which are more favorable than those in field settings) and the fact that all but one *P. falciparum *samples were obtained in travelers returning from Africa. In addition, the present study explored false positive Pv-pLDH lines exclusively in samples with high *P. falciparum *parasite densities, in line with the previous observations [2-4]. However, the inclusion of *P. falciparum *samples of lower parasite density and *Plasmodium *negative samples would have completed the picture. A prospective study in an area with *P. falciparum *and *P. vivax *coexistence should be performed to assess the relevance of the false positive Pv-pLDH lines in a field setting.

False-positive reactions of *P. vivax *samples with the HRP-2 line and the Pf-pLDH line have been described previously [24-26], but the presently observed false-positive Pv-pLDH lines in *P. falciparum *samples have only been reported anecdotally [2-4,22]. Among the panel of RDTs tested in the WHO and Foundation for Innovative New Diagnostics (FIND) study, there were two RDTs with a Pv-pLDH line: according to the tables, one of them generated false positive Pv-pLDH lines in 1.9% (6/316) of the *P. falciparum *samples tested.

Without exact knowledge of the antibodies targeting the Pv-pLDH antigen, it is difficult to explain the phenomenon, especially since false positive Pv-pLDH lines in the present study occurred not consistently among the different samples and brands. Weak cross-reactions may also be facilitated by slight differences in the composition of the nitrocellulose membrane and the diluent. It is known that several companies use the same pLDH antibodies in their RDT brands [27] and this may also be the case for Pv-pLDH: this is reflected by the identical product presentations and similar false positive rates of RDT brands numbers 6 and 7 and numbers 2 and 3 respectively. In the present study, there was no apparent relation between the false positive Pv-pLDH lines and the geographic origin of the infection, but it should be noted that mainly samples from sub-Saharan Africa were included.

The present study also revealed problems in RDT availability and communication with the supplier in the international market, as eight brands were not delivered despite several reminders. Furthermore, two of the RDTs that claimed to be *P. vivax*-specific by their label and name proved to detect pan-pLDH according to their package insert. Similar confusing names have been noted previously, also for the antibodies targeting aldolase [28].

## Conclusion

The occurrence of false positive Pv-pLDH lines in *P. falciparum *samples with high parasite densities was observed in six out of nine *P. vivax-*specific RDTs, in two-, three- and four-band RDTs. The false positive Pv-pLDH lines are of concern because the diagnosis of life-threatening *P. falciparum *malaria may be missed (two-band Pv-pLDH RDT), or the patient will be treated incorrectly with primaquine (three- or four-band RDT), which may cause severe hemolysis in patients with G6PD-deficiency. A prospective study in an area with *P. falciparum *and *P. vivax *coexistence should be performed to assess the relevance of the false positive Pv-pLDH lines in a field setting.

## List of abbreviations

FIND: Foundation of New Innovative Diagnostics; G6PD: Glucose-6-phosphate dehydrogenase; HRP-2: Histidine-rich protein 2; ITM: Institute of Tropical Medicine; *P.*: *Plasmodium*; pan-pLDH: pan *Plasmodium*-specific parasite lactate dehydrogenase; PCR: Polymerase chain reaction; Pf-pLDH: *Plasmodium falciparum-*specific parasite lactate dehydrogenase; pLDH: *Plasmodium*-specific parasite lactate dehydrogenase; Pv-pLDH: *Plasmodium vivax*-specific parasite lactate dehydrogenase; RDT(s): Rapid diagnostic test(s); WHO: World Health Organization.

## Competing interests

The authors declare that they have no competing interests.

## Authors' contributions

PG and JJ designed the study protocol. MvE and JvdE organized prospective sample collection. JM and PG carried out the test evaluations, LC performed PCR analysis. JM, PG and JJ analyzed and interpreted the results and JM and JJ drafted the manuscript. JM and PG performed statistical analysis. All authors read and approved the final manuscript.
